# Mismatch Between Preoperative Airway Assessment and Unanticipated Difficult Tracheal Intubation: A Retrospective Case–Control Study

**DOI:** 10.3390/healthcare14121619

**Published:** 2026-06-09

**Authors:** Chanatthee Kitsiripant, Wilasinee Jitpakdee, Maliwan Oofuvong, Pannawit Benjawaleemas, Nussara Dilokrattanaphichit, Wipharat Juthasantikul, Pannipa Phakam, Qistina Yunuswangsa, Polathep Vichitkunakorn

**Affiliations:** 1 Department of Anesthesiology, Faculty of Medicine, Prince of Songkla University, Hat Yai 90110, Thailand; 2 Department of Family Medicine and Preventive Medicine, Faculty of Medicine, Prince of Songkla University, Hat Yai 90110, Thailand

**Keywords:** airway management, difficult airway, intubation, patient safety, perioperative care, quality of care

## Abstract

**Highlights:**

**What are the main findings?**

- Most cases of unanticipated difficult tracheal intubation occurred in patients without obvious high-risk findings on routine preoperative airway assessment.
- Intubation difficulty became evident during laryngoscopy, characterized by poor visualization, repeated intubation attempts, and frequent escalation to advanced airway techniques.

**What are the implications of the main findings?**

- Routine preoperative airway assessment may not reliably identify all patients who subsequently experience difficult intubation.
- Difficult intubation may become apparent only during laryngoscopy despite apparently normal preoperative assessment findings.

**Abstract:**

**Background/Objectives:** Unanticipated difficult airway remains a critical patient safety concern in perioperative care. Despite routine preoperative assessment, difficult intubation may still occur in patients without obvious high-risk findings. This study aimed to evaluate perioperative factors associated with unanticipated difficult intubation and to examine the relationship between preoperative assessment and intraoperative intubation difficulty in routine clinical practice. **Methods:** This retrospective case–control study included adult patients undergoing general anesthesia with tracheal intubation between 2015 and 2020 at a tertiary care hospital. Unanticipated difficult intubation was defined as requiring ≥3 intubation attempts without documented preoperative suspicion of difficult airway. Patients with anticipated difficult airway or preoperative mechanical ventilation were excluded. A total of 95 cases and 429 controls were analyzed. Associations were explored using multivariable logistic regression. **Results:** Among 524 patients, cases more frequently had ASA physical status III and airway/neck/oral deformity. Notably, intubation difficulty became evident only at laryngoscopy, characterized by poorer visualization, increased intubation attempts (median 4 vs. 1), and frequent escalation to video laryngoscopy. Severe laryngoscopic views (Cormack–Lehane grade III–IV: 74.8% vs. 3.0%) were markedly overrepresented among cases. In multivariable analysis, ASA III and airway deformity remained independently associated with unanticipated difficult intubation. The model demonstrated modest discrimination (AUC 0.685). **Conclusions:** Unanticipated difficult intubation was uncommon but clinically important and frequently became apparent only during airway management. Although several associated factors were identified, routine bedside airway assessment alone may not reliably predict all cases of intraoperative difficult intubation. These findings highlight the limitations of routine bedside airway assessment in identifying all patients who subsequently experience difficult intubation and support the need for improved strategies to identify patients at risk.

## 1. Introduction

Unanticipated difficult tracheal intubation remains a critical challenge in perioperative practice and an important patient safety concern. Although the reported incidence of difficult tracheal intubation is relatively low, failure to promptly secure the airway can result in serious complications, including hypoxia, airway trauma, and mortality [1,2,3,4,5,6]. Data from the Perioperative and Anesthetic Adverse Events in Thailand (PAAd Thai) study reported a 2.3% incidence of unanticipated difficult intubation, highlighting the clinical relevance of this problem in routine practice [7]. Major airway complications continue to contribute substantially to perioperative morbidity and mortality [8,9].

Preoperative airway assessment traditionally focuses on anatomical predictors, including the Mallampati classification, thyromental distance, interincisor distance, and upper lip bite test [10]. However, the predictive performance of these bedside assessments remains limited, particularly when applied in isolation [11,12,13,14]. Emerging tools, such as airway ultrasound, may improve prediction but are not yet widely implemented in routine clinical practice [15,16,17]. Previous large observational studies have demonstrated that difficult airway management may still occur despite routine preoperative assessment and that prediction of airway difficulty in daily clinical practice remains imperfect [18,19]. Despite these advances, unanticipated difficult intubation continues to occur, suggesting that intubation difficulty cannot be fully explained by anatomical factors alone.

In routine clinical settings, this discrepancy between preoperative assessment and intraoperative airway difficulty represents a critical patient safety gap. Intubation difficulty may only become apparent during laryngoscopy, when visualization is inadequate and airway management must be rapidly escalated. In addition to anatomical factors, physiological conditions and perioperative context may influence airway management outcomes, further limiting the reliability of preoperative prediction. Therefore, this study aimed to evaluate perioperative factors associated with unanticipated difficult intubation and to examine the gap between preoperative airway assessment and intraoperative difficult intubation in routine clinical practice.

## 2. Materials and Methods

### 2.1. Study Design and Setting

This retrospective case–control study was conducted at a tertiary teaching hospital in Southern Thailand between January 2015 and December 2020. The study was reported in accordance with the Strengthening the Reporting of Observational Studies in Epidemiology (STROBE) guidelines.

### 2.2. Ethical Approval

Ethical approval was obtained from the Human Research Ethics Committee of the Faculty of Medicine, Prince of Songkla University (approval number 63-560-8-1). The requirement for informed consent was waived due to the retrospective nature of the study. All data were anonymized prior to analysis.

### 2.3. Study Population and Definitions

Adult patients (≥18 years) undergoing general anesthesia with endotracheal intubation for elective or emergency surgery were screened. Patients receiving preoperative mechanical ventilation were excluded.

Among the remaining eligible patients, 168 met the study definition of difficult intubation based on retrospective review of perioperative records. These patients were subsequently classified according to whether preoperative suspicion of difficult intubation had been documented before induction of anesthesia. Seventy-three patients with documented preoperative suspicion were classified as anticipated difficult intubation and excluded. The remaining 95 patients, who met the difficult intubation criteria without documented preoperative suspicion, were classified as unanticipated difficult intubation and constituted the case group.

Unanticipated difficult intubation was defined as requiring ≥3 intubation attempts using direct or video laryngoscopy in patients without documented preoperative suspicion, consistent with definitions used in prior observational studies [1,13,20].

### 2.4. Case and Control Selection

Cases were defined as patients with unanticipated difficult intubation. Controls were selected from patients without documented intubation difficulty during the same study period.

### 2.5. Airway Management Context

All patients included in the study underwent general anesthesia with tracheal intubation. Tracheal intubation was performed by anesthesia providers with varying levels of experience, including anesthesiology residents under supervision, nurse anesthetists, and attending anesthesiologists. The choice of intubation technique and airway device, including the use of direct or video laryngoscopy, was based on routine clinical judgment rather than a standardized airway management protocol.

### 2.6. Data Collection and Variables

Data were retrospectively extracted from electronic medical records and anesthetic records and curated into a structured dataset for analysis. Preoperative airway assessment variables routinely documented in clinical practice included Mallampati classification, thyromental distance, interincisor gap, upper lip bite test, neck mobility, dentition status, history of difficult airway, and the presence of airway/neck/oral deformity. Airway/neck/oral deformity referred to clinically documented structural abnormalities that could potentially affect airway alignment or laryngoscopic visualization, including congenital craniofacial abnormalities, prior head and neck surgery, cervical structural abnormalities, limited mouth opening, or visible anatomical distortion identified during routine perioperative assessment.

Collected demographic and clinical variables included age, sex, body mass index, ASA physical status, and relevant comorbidities (e.g., obstructive sleep apnea, tumors, trauma, and prior head and neck radiation). Perioperative variables included urgency and category of surgery (non-operating room anesthesia [NORA], neurosurgical/orthopedic surgery, ophthalmic/minor superficial surgery, otolaryngology surgery, thoracic/vascular surgery, and abdominal surgery), selected laboratory abnormalities potentially reflecting severe systemic illness or perioperative physiological instability (including coagulopathy and hypocalcemia), airway device usage, intubation provider, laryngoscopic view (Cormack–Lehane grade), number of tracheal intubation attempts, airway-related complications, and escalation of airway management techniques during intubation.

NORA procedures referred to anesthetic care provided outside the conventional operating room environment, including interventional radiology suites, gastrointestinal endoscopy units, cardiac catheterization laboratories, and other procedural areas requiring anesthesia care.

Airway management was performed according to routine clinical judgment rather than a standardized airway management protocol. Intubation attempts were performed by providers with varying levels of experience, including residents under supervision, attending anesthesiologists, and nurse anesthetists.

Video laryngoscopy was available during the study period but was primarily used as a rescue or escalation device rather than as a routine first-line intubation technique. Only variables consistently available in both cases and controls were included in the analysis. Additional supporting data regarding study variables are available in the Supplementary Materials (Table S1).

### 2.7. Statistical Analysis

Statistical analyses were performed using R version 4.3.1 (R Foundation for Statistical Computing, Vienna, Austria) [21]. Continuous variables are presented as medians with interquartile ranges and categorical variables as counts with percentages. Between-group comparisons were performed using Student’s t-test or Mann–Whitney U test for continuous variables and the chi-square test or Fisher’s exact test for categorical variables, as appropriate.

Univariable logistic regression was used to explore associations between candidate variables and unanticipated difficult intubation. Variables selected based on clinical relevance and univariable screening were entered into a multivariable logistic regression model. Collinearity was assessed prior to model construction. Results are reported as odds ratios (ORs) with 95% confidence intervals (CIs). A two-sided *p*-value < 0.05 was considered statistically significant. Model discrimination was evaluated using the area under the receiver operating characteristic curve (AUC). Given the retrospective observational design and limited number of outcome events, all analyses should be interpreted as exploratory and hypothesis-generating rather than confirmatory or intended for predictive model development.

Missing or undocumented variables were categorized as “unknown” where appropriate in descriptive analyses. Multivariable regression analyses were performed using complete case analysis without multiple imputation.

Sample size considerations were performed to estimate the approximate number of cases required for exploratory association analysis and feasibility assessment in the setting of a relatively low institutional incidence of unanticipated difficult intubation. An assumed odds ratio of 2.5 was selected based on effect sizes reported in prior observational airway studies. Under these assumptions, approximately 70 cases and 280 controls were estimated to provide 80% power at a significance level of 0.05.

## 3. Results

### 3.1. Study Cohort and Baseline Characteristics

**Figure 1:**
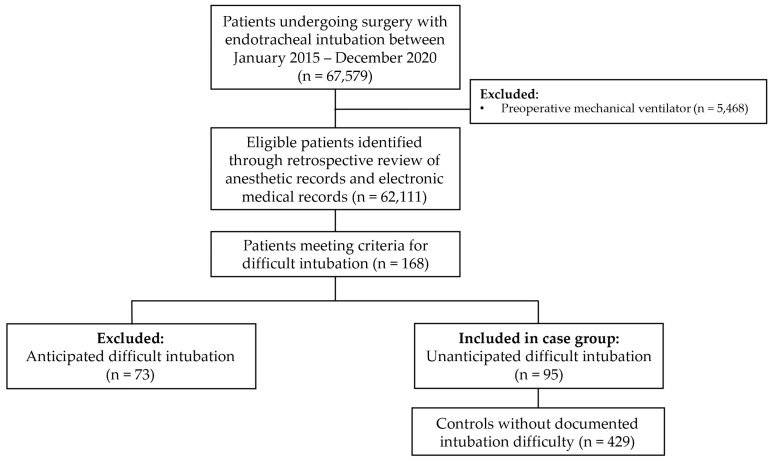
Flow diagram of the study.

A total of 524 patients were included in the final analysis, comprising 95 cases of unanticipated difficult intubation and 429 controls without preoperative suspicion of intubation difficulty (Figure 1). Baseline demographic characteristics are presented in Table 1.

Patients with unanticipated difficult intubation had a higher proportion of ASA physical status III (43.2% vs. 28.4%, *p* = 0.015) and airway/neck/oral deformity (8.4% vs. 2.6%, *p* = 0.012). Most routinely documented preoperative airway assessment variables were similar between groups, despite substantial differences in intraoperative laryngoscopic findings and intubation difficulty observed subsequently.

### 3.2. Intraoperative Airway Management and Laryngoscopic Findings

Intraoperative airway characteristics differed markedly between groups (Table 2). Unanticipated difficult intubation was characterized by substantially poorer laryngoscopic visualization and the need for escalation of airway techniques.

Compared with controls, cases demonstrated a markedly higher prevalence of Cormack–Lehane grade III–IV views (74.8% vs. 3.0%, *p* < 0.001), required more intubation attempts (median 4 [IQR 3–5] vs. 1 [IQR 1–1], *p* < 0.001), and more frequently required video laryngoscopy (GlideScope: 74.7% vs. 5.4%, *p* < 0.001). Emergency surgical airway was rare and did not differ significantly between groups.

Collectively, these findings indicate that intubation difficulty was primarily revealed intraoperatively rather than predicted preoperatively.

### 3.3. Multivariable Analysis

In multivariable logistic regression analysis (Table 3), ASA physical status III (adjusted OR 4.85, 95% CI 1.06–22.12, *p* = 0.042) and airway/neck/oral deformity (adjusted OR 4.09, 95% CI 1.43–11.66, *p* = 0.009) were independently associated with increased odds of unanticipated difficult intubation.

Male sex showed a trend toward higher risk but did not reach statistical significance (adjusted OR 1.56, *p* = 0.069). Other factors, including tumor, trauma, prior head and neck radiation, and metabolic abnormalities, were not independently associated with the outcome.

**Figure 2:**
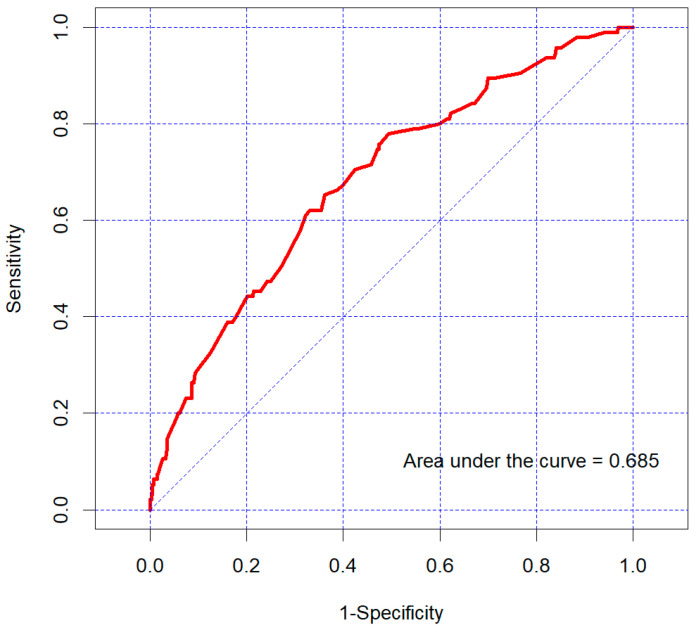
Receiver operating characteristic (ROC) curve of the multivariable logistic regression model.

The multivariable model demonstrated modest discriminative performance, with an area under the receiver operating characteristic curve (AUC) of 0.685 (Figure 2), corresponding to a sensitivity of 0.78 and specificity of 0.51.

## 4. Discussion

Unanticipated difficult intubation in this study was uncommon but clinically significant and occurred predominantly in patients without identifiable risk on routine preoperative assessment. This finding reinforces consistent evidence that commonly used bedside airway assessment tools have limited discriminative ability, particularly when applied in isolation [10,19,22,23]. The similarity of most routinely documented preoperative airway assessment findings between groups, contrasted with the marked differences observed in laryngoscopic view, intubation attempts, and the need for escalation to video laryngoscopy, illustrates the mismatch highlighted in the study title. Large observational studies have similarly demonstrated that difficult intubation may arise despite apparently normal preoperative findings [4,5], highlighting the limitations of current airway prediction strategies in routine clinical practice. Evidence from major airway audits further indicates that serious airway complications frequently occur in unanticipated scenarios and are influenced by human and system factors beyond anatomical predictors alone [9]. Collectively, these findings suggest that unanticipated difficult intubation should be viewed as an important patient safety issue that extends beyond the limitations of preoperative prediction alone.

In the present study, intubation difficulty was primarily revealed intraoperatively, characterized by poor laryngoscopic visualization and the need for escalation of airway techniques. The marked predominance of Cormack–Lehane grade III–IV views and repeated intubation attempts underscores that clinically relevant intubation difficulty often becomes evident only at the point of laryngoscopy. This observation highlights the limitations of preoperative assessment alone in identifying all cases of difficult intubation.

Among the evaluated factors, ASA physical status III and airway/neck/oral deformity were independently associated with unanticipated difficult intubation. Structural abnormalities, in contrast, directly impair airway alignment and visualization, providing a plausible anatomical basis for the observed association [24,25]. ASA III likely reflects increased physiological burden and reduced tolerance to airway manipulation, which may amplify the clinical impact of airway difficulty. Previous studies have demonstrated that higher ASA physical status is associated with an increased risk of difficult airway and perioperative complications [26]. Together, these findings support the concept that both physiological vulnerability and anatomical constraints contribute to airway management challenges.

Male sex showed a trend toward increased risk but did not reach statistical significance. Previous evidence suggests that sex-related anatomical differences may contribute to intubation difficulty; however, its predictive value remains inconsistent and context-dependent, and should therefore be interpreted with caution [25].

Other variables, including tumor, prior head and neck radiation, metabolic abnormalities, and operator-related factors, were not independently associated with the outcome after adjustment. Resident involvement was not independently associated with unanticipated difficult intubation after adjustment; however, this finding should be interpreted cautiously given the exploratory nature of the analysis and the limited number of outcome events. The absence of statistically significant association for head and neck tumors or prior radiation exposure should not be interpreted as evidence of absence of effect, as these factors are well-recognized factors associated with difficult airway management in the previous literature. The present findings may instead reflect limited statistical power, interactions among variables, study-specific characteristics, or residual confounding inherent to retrospective observational analysis. These findings suggest that such factors may play a context-dependent rather than primary role, or that their effects are mitigated within structured clinical environments.

Hypocalcemia and coagulopathy were included as exploratory perioperative variables because they may reflect severe systemic illness, trauma-related conditions, or physiological instability encountered during perioperative management. However, these variables are not established anatomical predictors of difficult intubation, and their observed associations should therefore be interpreted cautiously.

Importantly, repeated intubation attempts in retrospective routine clinical practice may not exclusively reflect anatomical airway difficulty but may also be influenced by operator experience, device selection, situational urgency, and clinical context. Therefore, the observed difficult intubation events likely represent an interaction among anatomical, physiological, operator-related, and system-related factors rather than purely anatomical airway difficulty alone. In addition, videolaryngoscopy during the study period was primarily used as an escalation or rescue technique rather than a routine first-line device, which may have influenced intubation patterns and escalation pathways observed in the present study.

The present study used a historical operational definition based primarily on repeated intubation attempts. Contemporary airway management concepts have evolved substantially and increasingly emphasize multidimensional airway difficulty, including physiological, situational, and human-factor components beyond anatomical intubation difficulty alone. Accordingly, the findings of the present study should be interpreted within the context of difficult tracheal intubation rather than the broader contemporary concept of difficult airway management.

Despite identifying associated factors, the overall discriminative performance of the multivariable model was modest (AUC 0.685), emphasizing the inherent limitations of prediction in this setting. No single variable or combination of routinely available clinical factors appears sufficient for reliable identification of patients at risk. These findings underscore the importance of recognizing the limitations of routine bedside airway assessment and maintaining vigilance throughout airway management [27]. The reported AUC should be interpreted cautiously, as the present analysis was exploratory and not intended for predictive model development or clinical risk stratification.

Given the retrospective observational design and relatively limited number of outcome events, the identified associations should be interpreted cautiously and considered exploratory and hypothesis-generating rather than causal.

This study has several limitations. First, the retrospective design may introduce information bias and precludes causal inference. Second, airway assessment variables were derived from routine clinical documentation and may not have been assessed or recorded with complete standardization, potentially limiting the ability to capture subtle anatomical or physiological factors relevant to difficult intubation. Third, the unmatched observational design may permit residual confounding related to patient comorbidities, provider experience, surgical characteristics, and airway management strategy. Airway management was not standardized and reflected routine clinical practice. Variability in provider experience, airway device selection, and escalation strategies may therefore have influenced the observed outcomes independently of patient-related airway anatomy. Furthermore, this was a single-center study conducted in a tertiary teaching hospital, which may limit generalizability to other settings.

Future prospective studies incorporating standardized airway assessment protocols, predefined documentation frameworks, and clearly defined airway management algorithms are warranted. Such studies may reduce information bias, improve consistency of data collection, and further clarify factors associated with unanticipated difficult intubation. Nonetheless, the large screened population and routine clinical setting provide clinically relevant insights into perioperative difficult intubation encountered in routine practice.

Overall, these findings highlight the limitations of routine bedside airway assessment in reliably identifying all cases of intraoperative difficult intubation. While preoperative airway assessment remains an essential component of perioperative evaluation, routine bedside assessment alone may not reliably identify all patients who subsequently experience difficult intubation.

## 5. Conclusions

Unanticipated difficult intubation is an uncommon but clinically important perioperative event. Although ASA physical status III and airway/neck/oral deformity were independently associated with unanticipated difficult intubation, most routinely documented preoperative airway assessment findings were similar between groups. These findings highlight the limitations of routine bedside airway assessment in identifying all patients who subsequently experience difficult intubation. While preoperative airway assessment remains an essential component of perioperative evaluation, it may not reliably identify all patients at risk of unanticipated difficult intubation.

## Figures and Tables

**Table 1:** Baseline characteristics and preoperative airway assessment of the study population.

Variables	Unanticipated Difficult Intubation (n = 95)	No Documented Intubation Difficulty (n = 429)	*p*-Value
**Demographics**			
Age (years), median (IQR)	54 (38.0–65.0)	52 (33.0–62.0)	0.262
Male sex	55 (57.9)	205 (47.8)	0.095
BMI (kg/m^2^), median (IQR)	23.5 (19.3–26.2)	22.5 (19.1–25.7)	0.572
ASA physical status			0.015
- I	2 (2.1)	32 (7.5)	
- II	51 (53.7)	271 (63.2)	
- III	41 (43.2)	122 (28.4)	
- IV	1 (1.1)	4 (0.9)	
**Airway-related conditions**			
Airway/neck/oral deformity *	8 (8.4)	11 (2.6)	0.012
Tumor (airway-related)	12 (12.6)	39 (9.1)	0.389
History of head and neck radiation	3 (3.2)	4 (0.9)	0.116
OSA/snoring	15 (15.8)	66 (15.4)	1
**Preoperative airway assessment**			
Mallampati class III-IV	10 (10.5)	20 (4.7)	0.084
Thyromental distance < 3 FB	3 (3.2)	15 (3.5)	0.992
Inter-incisor gap < 3 cm	7 (7.4)	16 (3.7)	0.290
Limited neck mobility	3 (3.2)	8 (1.9)	0.241
Upper lip bite test class III	1 (1.1)	3 (0.7)	0.529
**Perioperative factors**			
Coagulopathy	1 (1.1)	2 (0.5)	0.452
Hypocalcemia	1 (1.1)	0 (0)	0.181
**Surgical category**			0.448
Non-operating room anesthesia (NORA) **	13 (13.7)	53 (12.4)	
Neurosurgical/Orthopedic surgery	6 (6.3)	39 (9.1)	
Ophthalmic/Minor superficial surgery	12 (12.6)	75 (17.5)	
Otolaryngology (ENT) surgery	31 (32.6)	153 (35.7)	
Thoracic/Vascular surgery	11 (11.6)	33 (7.7)	
Abdominal surgery	22 (23.2)	76 (17.7)	

Data are presented as numbers (%); unless otherwise indicated. ASA, American Society of Anesthesiologists; BMI, body mass index; OSA, obstructive sleep apnea; IQR, interquartile range. * Airway/neck/oral deformity included clinically documented structural abnormalities potentially affecting airway alignment or visualization. ** NORA refers to procedures performed outside the conventional operating room environment, including interventional radiology suites, gastrointestinal endoscopy units, cardiac catheterization laboratories, and other procedural areas requiring anesthesia care.

**Table 2:** Intraoperative airway management and laryngoscopic findings.

Variables	Unanticipated Difficult Intubation (n = 95)	No Documented Intubation Difficulty (n = 429)	*p*-Value
**Laryngoscopic view (Cormack–Lehane)**			<0.001
- Grade I-II	23 (24.2)	407 (94.9)	
- Grade III-IV	71 (74.8)	13 (3.0)	
- Not recorded	1 (1.1)	9 (2.1)	
**Airway difficulty and attempts**			
Intubation attempts, median (IQR)	4 (3.0–5.0)	1 (1.0–1.0)	<0.001
**Airway device use**			<0.001
- Direct laryngoscopy	18 (18.9)	401 (93.5)	
- Video laryngoscopy	71 (74.7)	23 (5.4)	
- Advanced/rescue technique *	6 (6.3)	5 (1.2)	
**Provider characteristics**			
First-attempt provider (resident)	70 (73.7)	268 (62.5)	0.106
Experience < 5 years	90 (94.7)	400 (93.2)	0.631
**Airway-related complications**			
Emergency tracheostomy	1 (1.1)	1 (0.2)	0.331

Data are presented as numbers (%); unless otherwise indicated. IQR, interquartile range. * Advanced/rescue techniques include supraglottic airway devices, fiberoptic intubation, and other alternative airway strategies.

**Table 3:** Multivariable logistic regression analysis of factors associated with unanticipated difficult intubation.

Variable	Adjusted OR (95% CI)	*p*-Value
ASA physical status III (vs. I–II)	4.85 (1.06–22.12)	0.042
Airway/neck/oral deformity	4.09 (1.43–11.66)	0.009
Male sex	1.56 (0.97–2.53)	0.069
Tumor (airway-related)	1.99 (0.88–4.54)	0.100
History of head and neck radiation	3.85 (0.75–19.80)	0.106
Coagulopathy or hypocalcemia	4.99 (0.58–43.20)	0.145
Resident as first-attempt provider	1.57 (0.93–2.66)	0.094

OR = odds ratio, CI = confidence interval.

## Data Availability

The data presented in this study are available from the corresponding author upon reasonable request. The data are not publicly available due to privacy and ethical restrictions involving patient information.

## Supplementary Materials

The following supporting information can be downloaded at: https://www.mdpi.com/article/10.3390/healthcare14121619/s1, Table S1: Patient demographics and perioperative characteristics.

## Abbreviations

The following abbreviations are used in this manuscript:

ASA	American Society of Anesthesiologists
AUC	Area under the curve
CI	Confidence interval
IQR	Interquartile range
OR	Odds ratio
ROC	Receiver operating characteristic
