# Supplementary material for: Mismatch Between Preoperative Airway Assessment and Unanticipated Difficult Tracheal Intubation: A Retrospective Case–Control Study
Source: Healthcare (Basel). 2026 Jun 9;14(12):1619. doi: 10.3390/healthcare14121619 (PMC13299749; doi:10.3390/healthcare14121619)
Supplement: Supplementary file 1 [file healthcare-14-01619-s001.zip › healthcare-4327173-supplementary.pdf]

Supplement Table S1: Patient demographics and perioperative characteristics

Characteristics	Unanticipated difficult intubation (n = 98)	Non-difficult intubation (n = 294)	p-value
**Demographics**			
Age (years), median (IQR)	55 (38.0, 65.8)	56 (36.2, 64.0)	0.790
Male sex	56 (57.1)	146 (49.7)	0.243
BMI (kg/m^2), median (IQR)	23.3 (19,25.9)	22.7 (19.2,26)	0.210
ASA ≥ III	44 (44.9)	126 (42.9)	0.724
**Airway assessment**			
Preoperative suspected difficult airway	3 (3.1)	47 (16.0)	< 0.001
Mallampati class III-IV	10 (10.2)	26 (8.8)	0.911
Thyromental distance < 3 FB	3 (3.1)	8 (2.7)	0.832
Inter-incisor gap < 3 cm	7 (7.1)	16 (5.4)	0.824
Limit neck mobility	3 (3.1)	8 (2.7)	1
Upper lip bite test class III	1 (1.0)	3 (1.0)	0.681
**Modified Mallampati classification**			
1–2	79 (80.6)	239 (81.3)	
3–4	10 (10.2)	26 (8.8)	
unknown	9 (9.2)	29 (9.9)	
**Thyromental distance**			0.832
< 3 finger breaths	3 (3.1)	8 (2.7)	
3 finger breaths	64 (65.3)	181 (61.6)	
> 3 finger breaths	22 (22.4)	80 (27.2)	
unknown	9 (9.2)	25 (8.5)	
**Inter-incisor gap**			0.824
1–2 cm	7 (7.1)	16 (5.4)	
3–4 cm	82 (83.7)	251 (85.4)	

unknown	9 (9.2)	27 (9.2)	
**Limited neck flexion and extension**			1
No	94 (95.9)	284 (96.6)	
Yes	3 (3.1)	8 (2.7)	
Cannot evaluate	1 (1)	2 (0.7)	
**Upper lip bite test classification**			0.681
1	53 (54.1)	175 (59.5)	
2	26 (26.5)	75 (25.5)	
3	1 (1)	3 (1)	
unknown	18 (18.4)	41 (13.9)	
**Facial appearance or syndrome**			0.418
Normal	95 (96.9)	289 (98.3)	
Abnormal	3 (3.1)	5 (1.7)	
**Edentulous**			0.840
No	88 (89.8)	268 (91.2)	
Yes	10 (10.2)	26 (8.8)	
**Overbite**			0.250
No	97 (99)	294 (100)	
Yes	1 (1)	0 (0)	
**Previous history of difficult intubation and ventilation**			1
No	98 (100)	293 (99.7)	
Yes	0 (0)	1 (0.3)	
**Medical conditions**			1
No	93 (94.9)	278 (94.9)	
Yes	5 (5.1)	16 (5.4)	
**Congenital heart disease**			0.543
No	95 (96.9)	281 (95.6)	
Yes	3 (3.1)	13 (4.4)	
**Airway/neck/oral deformity**			0.483

No	90 (91.8)	276 (93.9)	
Yes	8 (8.2)	18 (6.1)	
**Foreign body aspiration**			0.438
No	97 (99)	293 (99.7)	
Yes	1 (1)	1 (0.3)	
**Infection: retropharyngeal abscess, epiglottitis, supraglottitis**			0.746
No	97 (99)	292 (99.3)	
Yes	1 (1)	2 (0.7)	
**Post-surgical procedure: thyroid, cervical vertebrae**			0.504
No	94 (95.9)	286 (97.3)	
Yes	4 (4.1)	8 (2.7)	
**OSA/Snoring**			0.875
No	83 (84.7)	245 (83.3)	
Yes	15 (15.3)	49 (16.7)	
**Tumors: thyroid, pharynx, larynx and tracheobronchus, esophagus**			0.501
No	86 (87.8)	265 (90.1)	
Yes	12 (12.2)	29 (9.9)	
**Trauma: face, neck**			0.643
No	96 (98)	290 (98.6)	
Yes	2 (2)	4 (1.4)	
**Burns (head, neck, face), smoke inhalation, massive burn**			0.261
No	96 (98)	292 (99.3)	
Yes	2 (2)	2 (0.7)	
**History radiation of head, neck**			0.697
No	95 (96.9)	288 (98)	
Yes	3 (3.1)	6 (2)	
**Laryngeal edema: angioedema, allergic, post rigid bronchoscopy**			0.746

No	97 (99)	292 (99.3)	
Yes	1 (1)	2 (0.7)	
**Coagulopathy and hypocalcemia**			0.095
No	95 (96.9)	292 (99.3)	
Yes	3 (3.1)	2 (0.7)	
**Surgical category**			0.448
Non-operating room anesthesia (NORA)	13 (13.7)	53 (12.4)	
Neurosurgical/Orthopedic surgery	6 (6.3)	39 (9.1)	
Ophthalmic/Minor superficial surgery	12 (12.6)	75 (17.5)	
Otolaryngology (ENT) surgery	31 (32.6)	153 (35.7)	
Thoracic/Vascular surgery	11 (11.6)	33 (7.7)	
Abdomen	22 (23.2)	76 (17.7)	
**First-attempt intubation provider**			0.106
Anesthesia instructors	9 (9.2)	31 (10.5)	
Anesthesiology residents	72 (73.5)	187 (63.6)	
Certified registered nurse anesthetists	3 (3.1)	28 (9.5)	
Nurse anesthetist students	14 (14.3)	48 (16.3)	
**Intubation experience (years)**			0.631
< 5	93 (94.9)	275 (93.5)	
5–10	3 (3.1)	13 (4.4)	
11–20	2 (2)	4 (1.4)	
> 20	0 (0)	2 (0.7)	
